# Expanding Biomaterial Surface Topographical Design Space through Natural Surface Reproduction

**DOI:** 10.1002/adma.202102084

**Published:** 2021-06-24

**Authors:** Steven Vermeulen, Floris Honig, Aliaksei Vasilevich, Nadia Roumans, Manuel Romero, Aysegul Dede Eren, Urnaa Tuvshindorj, Morgan Alexander, Aurélie Carlier, Paul Williams, Jorge Uquillas, Jan de Boer

**Affiliations:** ^1^ MERLN Institute Maastricht University Maastricht 6229 ER The Netherlands; ^2^ Department of Biomedical Engineering and Institute for Complex Molecular Systems Eindhoven University of Technology Eindhoven 5600 MB The Netherlands; ^3^ National Biofilms Innovation Centre Biodiscovery Institute and School of Life Sciences University of Nottingham Nottingham NG7 2RD UK; ^4^ Advanced Materials and Healthcare Technologies The School of Pharmacy University of Nottingham Nottingham NG7 2RD UK

**Keywords:** bacterial attachment, cell morphology, design space, microtopographies, natural surfaces, TopoChip

## Abstract

Surface topography is a tool to endow biomaterials with bioactive properties. However, the large number of possible designs makes it challenging to find the optimal surface structure to induce a specific cell response. The TopoChip platform is currently the largest collection of topographies with 2176 in silico designed microtopographies. Still, it is exploring only a small part of the design space due to design algorithm limitations and the surface engineering strategy. Inspired by the diversity of natural surfaces, it is assessed as to what extent the topographical design space and consequently the resulting cellular responses can be expanded using natural surfaces. To this end, 26 plant and insect surfaces are replicated in polystyrene and their surface properties are quantified using white light interferometry. Through machine‐learning algorithms, it is demonstrated that natural surfaces extend the design space of the TopoChip, which coincides with distinct morphological and focal adhesion profiles in mesenchymal stem cells (MSCs) and *Pseudomonas aeruginosa* colonization. Furthermore, differentiation experiments reveal the strong potential of the holy lotus to improve osteogenesis in MSCs. In the future, the design algorithms will be trained with the results obtained by natural surface imprint experiments to explore the bioactive properties of novel surface topographies.

## Graphical abstract

The topographical design space is immense, with many geometries existing. To explore this design space on cell behavior, the TopoChip is utilized with 2176 unique microtopographies. Nevertheless, phenotypical readouts are potentially lost due to an incomplete design space coverage. This coverage is expanded effectively in this work by including natural surfaces such as the red rose, leading to novel phenotypical discoveries.

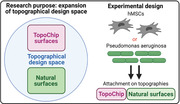

## 1 Introduction

Biological surfaces are interfaces between an organism and its environment and are the location where the organism deals with the chemical and physical reality of the outside world. Through evolution, biointerfaces have acquired functional characteristics to support survival, which can be of a chemical nature, as seen in plant waxes that decrease moisture loss^[^ ^1^ ^]^ and protect against UV radiation.^[^ ^2^ ^]^ On the other hand, topographical characteristics give surfaces fascinating and valuable properties. For example, the setae on gecko feet allow movement on smooth vertical walls^[^ ^3^ ^]^ and setae mimetics are used as dry and reversible adhesives for both robotic^[^ ^4^ ^]^ and biomedical applications.^[^ ^5^ ^]^ Superhydrophobicity, a material property observed on certain plant surfaces such as the holy lotus and red rose, is caused by hierarchical micro‐ and nanostructures and results in self‐cleaning surfaces.^[^ ^6^, ^7^ ^]^ Mosquitos use specialized superhydrophobic nanostructures on their eyes to prevent the nucleation of fog droplets^[^ ^8^ ^]^ and the tooth‐like scales on shark skin provide drag reduction, antibiofouling, and superoleophobicity, which protects sharks against oil spills.^[^ ^9^, ^10^ ^]^ Antimicrobial nanostructures on cicada wings may reduce the infection risk of implants,^[^ ^11^, ^12^ ^]^ and through inspiration from the *Nepenthes* pitcher plant, lubrication fluids cover micro/nanostructured medical devices with repellent and self‐cleaning surfaces.^[^ ^13^, ^14^, ^15^, ^16^ ^]^ These fascinating evolutionary and bioengineered structures demonstrate the richness in natural surface topographical bioactivity and begs research to use natural surface topographical design to improve the performance of materials for industrial and clinical applications.

Surface topography is used in tissue engineering to guide cell behavior in vitro and in vivo to enhance the biocompatibility of medical devices.^[^ ^17^ ^]^ Research in recent years highlighted the challenges to find optimal topographies for specific applications due to the very large design space, which we define as the universe of surface architectures. This ranges from randomly introduced roughness^[^ ^18^ ^]^ to designed groove patterns.^[^ ^19^ ^]^ Additionally, topographies exist as pillars^[^ ^20^ ^]^ or complex geometries,^[^ ^21^, ^22^ ^]^ while curvature provides convex and concave shapes.^[^ ^23^ ^]^ All these structures, found both in micro‐ and nanometer dimensions, are known to affect the cells that are in contact with them.

The large topographical design space complicates the quest for the optimal topography for a specific application. To this end, high‐throughput topography screening (HTS) platforms were developed and many novel bioactive surfaces have been discovered.^[^ ^24^ ^]^ Examples include the microgrooved polyimide chip^[^ ^19^, ^25^ ^]^ and the integrated mechanobiology platform (IMP)^[^ ^26^ ^]^ containing grooves and ridges in different dimensions. The IMP platform also contains topographical structures, similar to those of the BioSurface Structure Array with 504 unique combinations of circles, squares, and rectangles.^[^ ^27^, ^28^ ^]^ Similarly, the multiarchitecture chip platform with 18 distinct surface grooves, pillars, and pits enhances surface topography diversity.^[^ ^29^, ^30^ ^]^ Our group has developed the TopoChip platform with 2176 unique microtopographies composed of circles, triangles, and rectangles in different sizes and combinations.^[^ ^21^ ^]^ The TopoChip allowed us to identify topographies that promote tenocyte phenotype,^[^ ^31^ ^]^ osteogenic differentiation,^[^ ^17^ ^]^ and TGF‐β signaling sensitivity.^[^ ^32^ ^]^ All these platforms used as high‐throughput screening to study interactions between cells and topographies have provided a wealth of information for regenerative medicine applications.

However, HTS platforms still have their limitations because their design strategy only covers a small part of the whole design space. For example, structures in both nano‐ and micrometer dimensions are rarely present in the same platform, and variable roughness levels are not included in a high‐throughput setting. Human‐made designed surface structures are frequently presented in an organized pattern, yet disorder also profoundly influences cell behavior.^[^ ^33^, ^34^ ^]^ The bottleneck in producing a more diverse spectrum of surface topographies is not in the in silico design possibilities, where algorithms such as neural networks could aid the design and fine‐tuning of surface topographies,^[^ ^22^ ^]^ but rather the technical limitations in surface topography manufacturing. Even though state‐of‐the‐art techniques such as two‐photon stereolithography can handle the fabrication of complex shapes,^[^ ^35^ ^]^ its limited writing speed remains unsuitable for high‐throughput applications. On the other hand, photolithography is mostly a 2D technique unsuitable for introducing design variability in height (*z*‐axis) of complex topographies produced at a large scale. We hypothesize that we can increase design space by using natural surfaces as a mold and solvent casting as a microfabrication technique to replicate them into materials of interest, in our case, tissue culture polystyrene (PS). Many reports describe the replication of natural surfaces^[^ ^36^, ^37^, ^38^, ^39^, ^40^ ^]^ and in this work, we used a quantitative approach for comparing design space coverage by artificial and natural surfaces.

We investigated the combination of multiple natural surfaces in one platform to create a novel architectural design sub‐space not commonly found in artificial platforms. We sampled a diverse set of 26 plant and animal surfaces, reproduced their structures in polystyrene, and investigated their potential for controlling cell behavior. We demonstrated that the structural diversity from the natural surfaces surpasses that of the microtopographical TopoChip platform and show novel stem cell and bacterial bioactivity in this array of natural surface topographies.

## 2 Results and Discussion

### 2.1 Natural Surface Architectures Exhibit a Wide Design Variety

**Figure 1:**
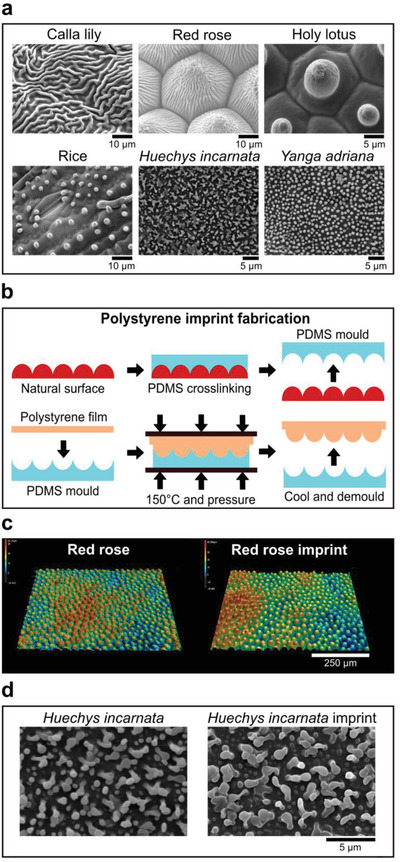
Natural surfaces exhibit a large topographical variety. a) SEM images of leaves and insect wings utilized in this study. b) Fabrication scheme to imprint natural surface designs onto polystyrene. Once the PDMS mold has been crosslinked, a polystyrene film is placed on top of it, pressed, and heated to transfer the natural surface topography to polystyrene. c) Leaf imprints can be transferred to polystyrene with high fidelity, as seen in the profilometric images of red rose and its imprint counterpart. d) Insect wing imprints can be transferred to polystyrene with high fidelity, as seen in the SEM images of *Huechys incarnata* and its imprint counterpart.

We selected 16 plant and ten insect surfaces with a diverse set of surface properties based on reported phenomena such as superhydrophobicity, antifouling, or light reflection (see Figures S1 and S2, Supporting Information). Observation of the unprocessed specimens using scanning electron microscopy revealed an exciting variety in surface topography. For example, the calla lily petal surface exhibited interconnected cuticular folds with ridges of 1 µm in height (**Figure** **1**a, top row, left panel), which is very different from the petal surface of the red rose, which has parallel‐aligned hierarchical structures of 20 µm high micropapillae and nanofolds (Figure 1a, top row, center panel). Holy lotus, known for its superhydrophobicity, has heptagonal inclinations, a nano rough surface, and convex micro curvature resulting in a 10 µm high pillar in the center (Figure 1a, top row, right panel). On the rice surface, we found pillar structures with longitudinal ridges (Figure 1a, bottom row, left panel). The *Huechys incarnata* wing is an interesting case, where merged pillars are present, which give rise to a variable structure size between 500 nm and 5 µm in diameter (Figure 1a, bottom row, center panel). Variable pillar sizes can be found on cicadas such as the *Yanga adriana* (Figure 1a, bottom row, right panel). We classified plant surfaces into five groups: a) cuticular folds with low elevation, b) cuticular folds with high elevation, c) oriented structures, d) complex structures, and e) microroughness (Figure S3, Supporting Information). Three classes were observed on animal surfaces: a) nanopillars, b) pits, and c) curved surfaces (Figure S4, Supporting Information).

### 2.2 Natural Surface Architectures Can Be Imprinted with High Fidelity into Polystyrene

We chose polystyrene as reference chemistry in which to compare cell–material interaction.^[^ ^41^, ^42^ ^]^ In order to transfer the natural surface structures into PS, we used a relatively easy and fast technique that only requires the use of glass slides, binder clips, and a conventional oven (Figure 1b).^[^ ^43^ ^]^ Poly(dimethylsiloxane) (PDMS) was cast upon the natural surface, after which it was cured at room temperature for 24 h. The PDMS containing the negative imprint of the natural topographies was peeled off the natural surface (Figure 1b, top row and Figure S5A, Supporting Information) and a “sandwich” was created containing glass slides, Teflon sheets, the PDMS imprint, and a polystyrene sheet (Figure 1b, bottom row and Figure S5B, Supporting Information). The construct was placed in the oven, and after 1 h, the construct was cooled to room temperature, and the PS imprint peeled off the PDMS.

We next compared the profilometric images of the PS imprints and original specimens, and using high‐resolution microscopy, we found that the imprints were successfully transferred with high fidelity from natural surfaces into PS. Examples are provided for the leaves of the red rose (Figure 1c) and *Huechys incarnata* (Figure 1d). Sub‐micrometer structures present on the cuticular folds of the red rose and holy lotus were also successfully replicated, although we do observe for the holy lotus imprint an increase of surface roughness (Figure S6A,B, Supporting Information).

Then, we assessed as proof of concept the superhydrophobic properties of both the red rose and holy lotus imprints by measuring the water contact angle. No significant differences were seen between the natural and PS imprints (Figure S7, Supporting Information). We also measured the water contact angle of other natural PS imprints and found that the majority of the imprints induced higher contact angles compared to flat PS, with the holy lotus demonstrating the highest hydrophobic property (Figure S8A, Supporting Information). Similarly, we measured the water contact angle of 26 randomly chosen microtopographical PS imprints from the TopoChip library and found an increase in the water contact angle across the imprints compared to flat PS (Figure S8B, Supporting Information). Of interest, we found that high contact angle corresponds to high pattern density and a lower contact angle with lower pattern density (Figure S8C, Supporting Information). The rose petal effect, resulting in the pinning of a water droplet,^[^ ^7^ ^]^ was demonstrated by inverting and tilting the imprint. The lotus effect, which is characteristic of the rapid rolling of water droplets on the surface, was also seen on the PS holy lotus imprint (data not shown). These examples demonstrate that also the physical properties of natural topographies can be replicated on PS.

### 2.3 Natural Surface Architectures Occupy a Different Part of the Design Space and Induce Distinct Cell Morphology Compared to TopoChip Microtopographies

**Figure 2:**
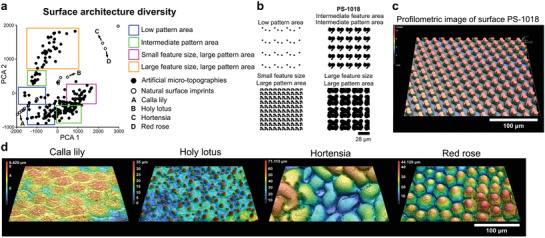
Natural surfaces expand the topographical design space. a) Principal component analysis of the TopoChip and natural surface imprints reveals distinct clustering representing different material designs. b) In silico design of artificial microtopographies of different clusters. c) Profilometric image of surface PS‐1018, exhibiting an intermediate pattern area. d) Representative examples of profilometric images of natural surface imprints.

The objective of this study was to determine whether natural surface topographies occupy a different part of the design space than our previously established library of randomly generated TopoChip topographies.^[^ ^21^, ^44^ ^]^ We quantified the dimensional features of 199 randomly sampled TopoChip surfaces and the natural PS surface imprints based on the design file and interferometry data, respectively, and plotted the differences using principal component analysis (PCA) (**Figure** **2**a).^[^ ^21^ ^]^ PCA is a dimension reduction technique that condenses multidimensional data into fewer features and allows a visual representation of the variation of the principal components. Each data point in the PCA plot represents a single surface and the distance between dots represents their similarities. Interestingly, TopoChip topographies formed four separate clusters (Figure 2b): designs with: a) low‐density pattern areas, large pattern areas composed of either: b) small or c) large features, and d) intermediate pattern areas. An example of a surface design with an intermediate pattern area can be found in Figure 2c. On the other hand, natural surfaces formed a fifth cluster (Figure 2a represented by points (A) to (D) with topographies of variable size and complex features and patterns (Figure 2d). These observations demonstrate that natural surfaces represent an unexplored part of the topographical design space.

**Figure 3:**
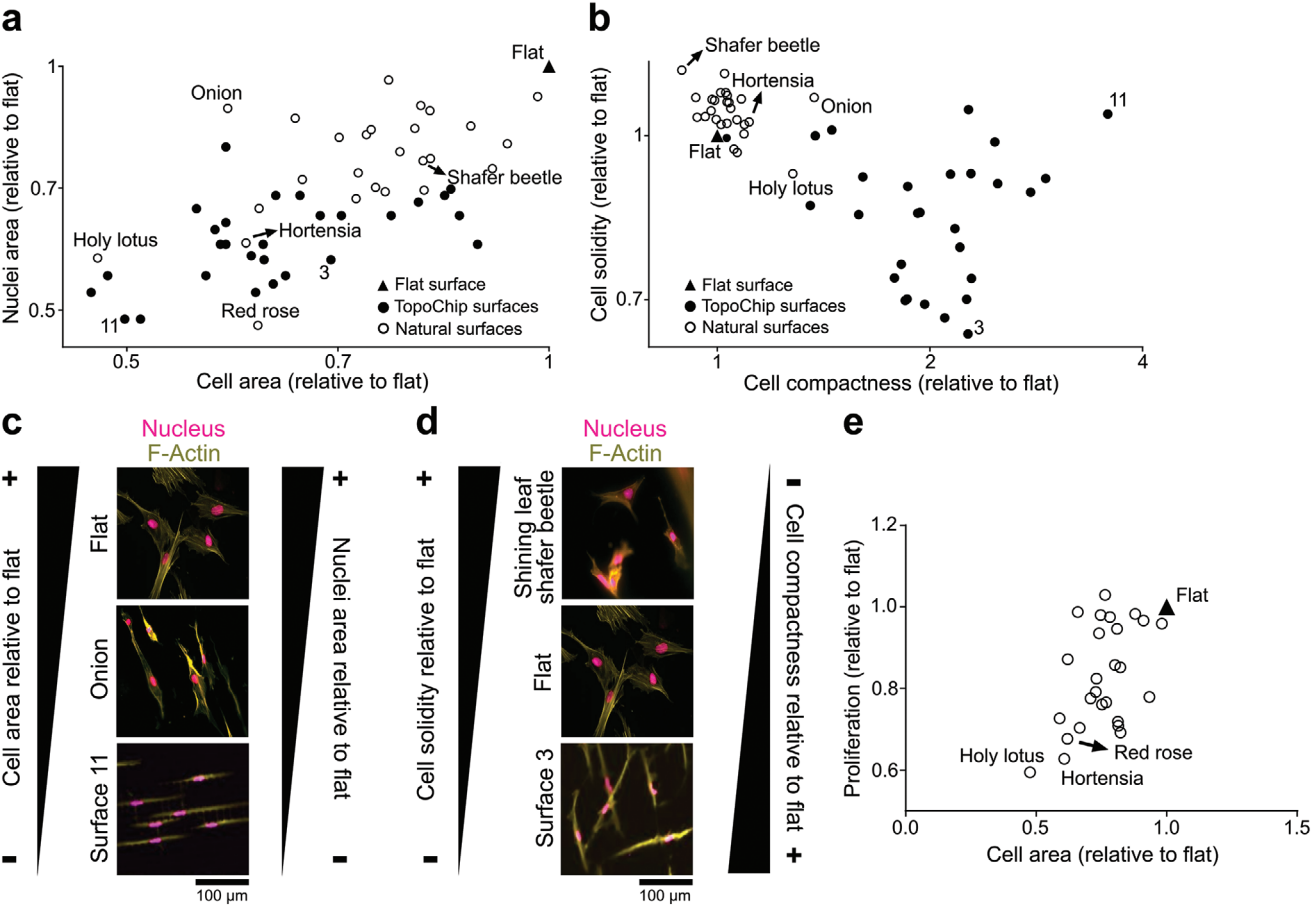
TopoChip and natural surface imprints have a different quantitative effect on the shape of MSCs. a) Cell and nucleus area quantification of MSCs on TopoChip and natural surface imprints. b) Cell compactness and solidity quantification of MSCs on TopoChip and natural surface imprints. c) Visual representation of the change in cell and nucleus area of MSCs on TopoChip and natural surface imprints. d) Visual representation of the change in cell compactness and solidity of hMSCs on TopoChip and natural surface imprints. F‐Actin is stained with phalloidin (yellow) and nuclei with Hoechst (magenta). e) Proliferation rate of MSCs on natural surface imprints as a function of cell area. The proliferation rate of MSCs decreases with reduced cell size.

Cell shape and surface topography are highly correlated^[^ ^45^, ^46^, ^47^ ^]^ and we wondered if a more extensive design space also leads to unobserved cell shape features. To this end, we seeded human mesenchymal stem cells (hMSCs) onto the PS imprints of 26 natural surfaces and onto 28 TopoChip topographies selected to capture the whole range of cell shape variation on the TopoChip.^[^ ^48^ ^]^ F‐actin and DNA were immunolabeled, and quantitative information of cell and nucleus area, and cell compactness and solidity data were extracted for all surfaces (**Figure** **3**).^[^ ^49^ ^]^ In Figure 3a, the effect of topography on MSC cell and nucleus area is compared relative to MSCs on flat PS. The further a data point is from the unit coordinates and closer to the plot origin, the smaller is the cell and nucleus area. In general, MSCs seeded on natural surfaces showed larger cell and nucleus areas compared to the size of cells on the TopoChip. We found that the holy lotus, red rose, and hortensia induce the smallest cell sizes of all natural surface topographies.

Cell compactness relates to cell elongation and cell solidity is inversely correlated to cell branching and radial filopodia protrusion. A high compactness value indicates a more elongated cell, and a high solidity value indicates that the cell is less branched and has fewer filopodial protrusions extending radially. We observed that cells on natural surfaces tend to have less cell branching and radial filopodia protrusion, with most cells on natural surfaces exhibiting solidity values of more than 1 (Figure 3b). TopoChip surfaces typically induce compactness values of more than 1, as seen on the TopoChip surface 3, where cells were more elongated and branched than cells on natural surfaces. For the natural surfaces, the onion demonstrated evident elongated characteristics. Representative images of the natural and TopoChip imprints can be found in Figure 3c,d. We further found that proliferation rate can be correlated to cell area (Figure 3e). MSCs on all natural surfaces showed a decrease in proliferation rate when compared to flat culture surfaces. This result is comparable with a previous report from our group that indicated a negative effect on proliferation rate when MSCs were cultured on microtopographies.^[^ ^50^ ^]^ In general, the analysis of cell compactness and solidity between artificially designed and natural surfaces shows that natural topographies induce cell morphologies distinct from those observed on the TopoChip.

### 2.4 Natural Surface Architectures Affect the Organization of Actin Fibers and Focal Adhesions and Alter the Differentiation Potential of MSCs

**Figure 4:**
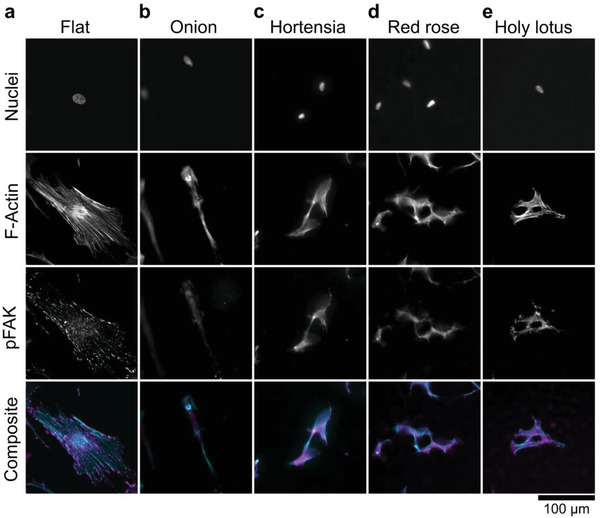
Natural surface imprints induce substantial alterations in the distribution of cytoskeleton components (F‐actin) and focal adhesions. a) MSCs cultured on flat exhibit visible stress fibers with dot‐like focal adhesions. b) MSCs cultured on the onion lose stress fibers formation. c,d) Both the hortensia and red rose exhibit diffuse F‐actin architecture with little focal adhesions. e) Small F‐actin stress fibers are visible on MSCs cultured on the holy lotus with dot‐like focal adhesions. The nucleus is stained with Hoechst 33258, F‐Actin is stained with phalloidin (cyan) and pFAK immunolabeled with a primary antibody (magenta).

To investigate if the natural surface topographies can alter F‐Actin characteristics and focal adhesion activation, we supplemented the F‐Actin immunolabel with phosphorylated focal adhesion kinase (p‐FAK) staining (**Figure** **4**). MSCs cultured on flat surfaces exhibit a strong presence of F‐Actin stress fibers and mature focal adhesion sites (Figure 4a). MSCs grown on the onion surface demonstrated a reduction in F‐Actin stress fibers and the presence of immature focal adhesions at the periphery of the cell (Figure 4b). Diffuse F‐Actin stress fibers were observed in MSCs grown on the hortensia, and red rose, with few focal adhesions (Figure 4c,d). Short but visible F‐Actin stress fibers were present in MSCs cultured on the holy lotus surface (Figure 4e). A strong presence of immature dot‐like focal adhesions in MSCs cultured on the holy lotus surface. It has been demonstrated that both actin/myosin tension and FAK signaling are implicated in cell differentiation.^[^ ^51^, ^52^ ^]^ Therefore, these observations can shed some light in determining how natural topographies guide MSC differentiation.

**Figure 5:**
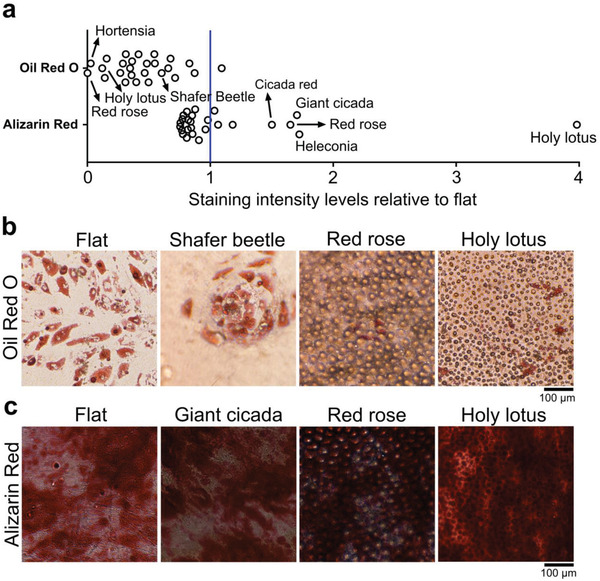
Natural surface imprints alter the differentiation potential of MSCs. a) Quantification of Oil Red O and Alizarin Red staining relative to flat surface revealed a reduction in the adipogenic potential of MSCs on natural surfaces. Conversely, surfaces such as the holy lotus and red rose strongly promote osteogenesis. b) Representative images of the Oil Red O staining. Note an increase in adipogenesis in the pit‐like structures of the shining beetle. c) Representative images of the Alizarin Red staining. The holy lotus induced intense mineralization.

Next, we investigated if natural topographies have the ability to alter the differentiation potential of MSCs (**Figure** **5**). We cultured MSCs for 21 d on natural topographies with either adipogenic or osteogenic media and evaluated mineralization through Alizarin Red and adipogenesis through Oil Red O (Figure 5a). We found that the majority of the surfaces do not promote adipogenesis (staining intensity levels between 0 and 1), with no observable fat droplets on the red rose, and a small number of droplets on the holy lotus surface (Figure 5b). However, we noticed fat droplet deposition within the pits of the shining beetle surface. As to osteogenic differentiation, we found the red rose and especially the holy lotus surfaces induced strong osteogenic MSC differentiation (Figure 5c). We also noticed improved osteogenic differentiation on the heliconia, the cicada red and the giant cicada surfaces. The strong potential of the holy lotus surface to promote mineralization was also described in osteosarcoma MG63 cells seeded on holy lotus imprints of polycaprolactone.^[^ ^53^ ^]^ It is yet to be confirmed whether it is surface roughness, the pillars, or both combined that create the hierarchical structure of the holy lotus, that induces osteogenesis.

### 2.5 Natural Surface Topographies Induce Distinct Profiles of *Pseudomonas aeruginosa* Colonization

**Figure 6:**
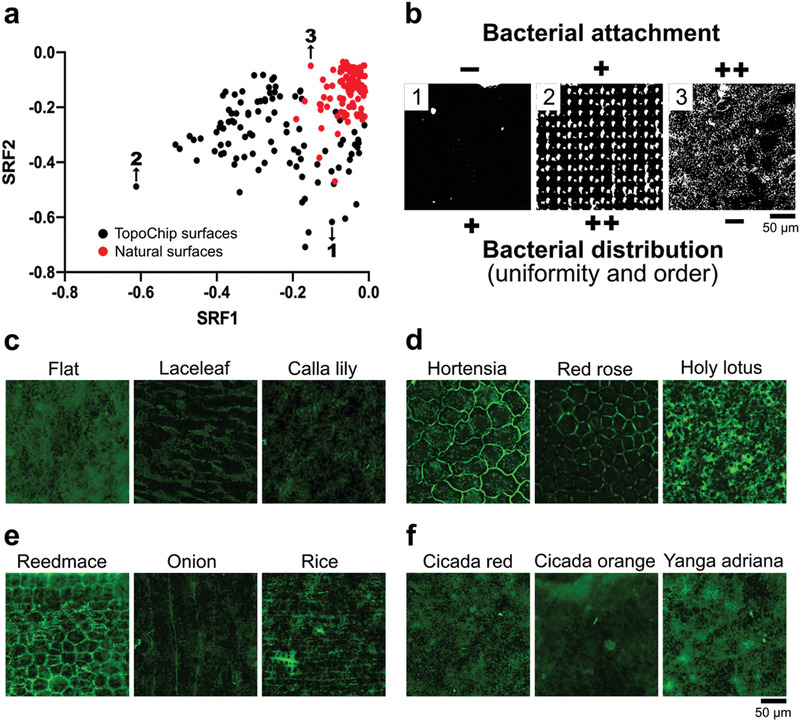
TopoChip and natural surface imprints have a different quantitative effect on the attachment distribution of *P. aeruginosa*. a) Spatial relationship feature plot of *P. aeruginosa* on TopoChip (black dots, points 1 and 2) and natural surface imprints (red dots, point 3). b) Visual representation of the bacterial attachment and distribution of *P. aeruginosa* on TopoChip (images 1 and 2) and natural surfaces (image 3). (−) = low, (+) = medium, and (++) = high. c) Representative bacterial attachment images on the natural surface imprints with low height cuticular folds reveal that attachment occurs in various patterns where the bacteria preferably attach next to the cuticular folds and inside the groove regions. d) This is further emphasized by plant surfaces that exhibit high cuticular folds, where bacteria preferably attach to the groove regions. Only the red rose demonstrated a general reduction in bacterial attachment. e) For orientated structures, bacteria prefer to attach in regions with grooves. f) The cicada surfaces and *Yanga adriana* exhibited bacterial attachment similar to flat surfaces. Bacteria were fluorescently labeled through SYTO9.

Bacteria colonize diverse natural and artificial surfaces forming biofilms that confer protection against environmental stresses.^[^ ^54^ ^]^ Such biofouling is highly problematic in both industrial and medical contexts. Bacterial biofilm formation can be prevented by incorporating toxic biocidal agents by modifying surface chemistry or surface topography.^[^ ^55^, ^56^ ^]^ Several studies have shown that bacterial attachment can be controlled using patterned surfaces featuring repeating topographical elements of sizes ranging from nanometers to micrometers. Some of these patterned surfaces have been based on nonfouling natural surfaces, including shark skin, plant leaves, and insect wings.^[^ ^55^, ^57^ ^]^ However, to our knowledge, no systematic high throughput screens have yet been published. Hence, we compared the patterns of bacterial attachment to the 26 natural surfaces against TopoChip imprints. *P. aeruginosa* was chosen as the model bacterium as it is an environmentally ubiquitous microorganism that readily adheres to natural and engineered surfaces. This microorganism is highly problematic in a clinical infection context as it forms multiantibiotic‐resistant biofilms on implanted medical devices.^[^ ^58^ ^]^ The attachment distribution profiles of *Pseudomonas aeruginosa* after 4 h growth on the natural surfaces were compared with those observed on 100 randomly sampled TopoChip topographies using the texture parameters in CellProfiler software. The spatial relation features 1 and 2 are shown in **Figure** **6**a, which demonstrated that the natural surfaces occupy a clearly different area of distribution to the TopoChip surfaces. In general, we noted more uniform and ordered bacterial distribution on the TopoChip surfaces (Figure 6b).

In specific natural surfaces like the lace leaf and calla lily, we noticed that cuticular folds prevented bacterial attachment compared to flat surfaces (Figure 6c). On the hortensia and red rose surfaces, bacteria attached mainly on the grooves next to the folds (Figure 6D). The holy lotus surface, however, showed significantly high bacterial attachment.

Similarly, high bacterial attachment and distribution were observed in the reedmace and rice surfaces (Figure 6E). In contrast, the onion surface exhibited reduced bacterial attachment, with preferable bacterial adhesion along its grooves.

As for the insects’ surfaces, we observed similar bacterial attachment as on flat surfaces (Figure 6F), with only a mild decrease on the cicada orange surface.

Finally, our ongoing efforts are directed to extract individual features of artificial and natural surfaces to induce a specific cell response and use this Topo‐natural‐chip, in combination with our newly developed ChemoTopoChip,^[^ ^59^ ^]^ as a screening platform for studying the patient‐specific innate inflammatory response to implants and coating materials.

## 3 Conclusion

We have transferred features of natural surfaces to cell culture platforms to increase the topographical design space. We demonstrated that natural surfaces: i) could be transferred with high fidelity in polystyrene, ii) occupy an unexplored area of topographical design space relative to the TopoChip, iii) uniquely alter morphological characteristics of human mesenchymal stem cells, iv) alter their differentiation potential, and v) affect the spatial distribution of *Pseudomonas aeruginosa* attachment. With natural surfaces, we can step out of the limitations of the TopoChip and bring new and unique unit features into the design algorithm. In this work, we used only 26 natural surfaces, a minuscule subset of all the plants and insects on the planet. We envision the replication of plants and insects topographies in the most biodiverse regions on Earth.^[^ ^60^ ^]^ For instance, we would like to explore topographical design space at the Tiputini Biodiversity Station in the Ecuadorian Amazon rainforest, one of the most biodiverse tropical forests in the world,^[^ ^61^, ^62^, ^63^, ^64^ ^]^ and scan natural surfaces with a portable profilometer based on interferometry to obtain a digital representation of natural surfaces.^[^ ^65^ ^]^ Deep learning algorithms developed in‐house^[^ ^66^, ^67^ ^]^ and in ref. ^[^ ^68^ ^]^ can decouple complex topographical information of natural surfaces and provide guides in selecting surfaces that cover uncharted topographical territory. Two‐photon lithography can then be used to generate the surfaces and analyze their bioactive properties. Our work is ongoing to unveil advanced and nature‐inspired surfaces with unique bioinstructive properties that could be used in disease and patient‐specific regenerative medicine applications.

## Conflict of Interest

The authors declare no conflict of interest.

## Supporting information

Supporting Information

## Data Availability

Research data are not shared.
