# Supplementary material for: Expanding Biomaterial Surface Topographical Design Space through Natural Surface Reproduction
Source: Adv Mater. 2021 Jun 24;33(31):2102084. doi: 10.1002/adma.202102084 (PMC11468538; doi:10.1002/adma.202102084)
Supplement: Supplementary file 1 — Supporting Information [file ADMA-33-2102084-s001.pdf]

© 2021 Wiley-VCH GmbH

ADVANCED MATERIALS

Supporting Information

for *Adv. Mater.,* DOI: 10.1002/adma.202102084

Expanding Biomaterial Surface Topographical Design Space through Natural Surface Reproduction

*Steven Vermeulen, Floris Honig, Aliaksei Vasilevich, Nadia Roumans, Manuel Romero, Aysegul Dede Eren, Urnaa Tuvshindorj, Morgan Alexander, Aurélie Carlier, Paul Williams, Jorge Uquillas, and Jande Boer**

Supporting Information

Expanding Biomaterial Surface Topographical Design Space through Natural Surface Reproduction

*Steven Vermeulen, Floris Honig, Aliaksei Vasilevich, Nadia Roumans, Manuel Romero, Aysegul Dede-Eren, Urnaa Tuvshindorj, Aurélie Carlier, Paul Williams, Jorge Alfredo Uquillas, Jan de Boer**

Corresponding author
*E-mail: j.d.boer@tue.nl

Experimental Section

Fabrication of artificial micro-topographies. A detailed description describing the fabrication of the micro-topographical surfaces can be found elsewhere.[69] In brief, the inverse pattern was etched on a silicon wafer by directional reactive ion etching (DRIE). To facilitate subsequent demolding procedures, the silicon wafer was coated with a layer of perfluorooctyltrichlorosilane (FOTS, Sigma-Aldrich). Polydimethylsiloxane (PDMS; Sylgard® 184 Elastomer Kit, Dow Corning) in a 10:1 silicon/curing agent ratio (w/w) was cured on the waver at 85 ºC for 6 h to generate a positive mold. OrmoStamp hybrid polymer (micro-resist technology Gmbh) was subsequently poured on the PDMS imprint together with a borofloat waver containing a layer of Ormoprime (micro-resist technology Gmbh). UV treatment allowed the cross-linking of a final negative Ormostamp imprint, which is used as a template for the hot embossing step at 140 °C for 10 min to generate a polystyrene imprint.

Fabrication of natural surface topographies. Dried insects used in this study were purchased from the supplier “The Bugmaniac” (http://www.thebugmaniac.com). The Holy Lotus (Nelumbo nucifera), Rice (Oryza sativa), Plaintain Lily (Hosta sp.), and Hardy Canna (Thalia dealbata) were kindly donated from the botanical garden “Hortus Botanicus” of Leiden (https://www.hortusleiden.nl). Other plants were purchased at the local flower shop. Replication of natural surfaces was achieved through a two-step fabrication process. [43] The first step included the fabrication of a polydimethylsiloxane (PDMS) mold, which contains the negative imprint of the natural surface. A sample of the fresh natural surface of choice was fixed onto a petri dish using

double-sided adhesive tape (Double Fix, Bison). The silicon resin and curing agent (Sylgard® 184 Elastomer Kit, Dow Corning) were mixed in a 10:1 ratio (w/w) and cast onto the natural surface. Subsequently, the liquid mixture was degassed and cured for 48 h at room temperature to prevent heat damage. Finally, the solid PDMS mold was separated from the natural surface. In the second step, the PDMS mold served as a template for transferring the structures into PS using hot embossing. To achieve this, an assembly was made in which a 190 µm thick PS film was placed on the PDMS mold clamped between two Teflon sheets and microscope slides to apply constant pressure. To cross the glass-transition of the PS film, the entire system was inserted in an oven for 1h at 150 °C. Afterward, the assembly was removed from the oven and cooled down to room temperature to allow the PS film to solidify. Finally, the PS surface with transferred surface topography was carefully peeled from the PDMS mold.

Scanning electron microscopy imaging. Scanning electron microscopy was used for examination of the surface topography of natural surfaces and quality control of their respective PS replicas. Preparation of fresh natural samples consisted of fixation (2% glutaraldehyde in 0.1M cacodylate buffer) for 1hr. Subsequently, the samples were washed three times in cacodylate (0.1M) before dehydration by immersion in a graduated series of ethanol in water (50%, 70%, 90%, 100%). Then, samples were critical point dried (EM CPD300, Leica) using 15 exchange cycles with slow gas out and heating speed settings. All samples were mounted on SEM stubs using carbon conductive adhesive tape. Finally, samples were sputter-coated with gold (Sputter Coater 108auto, Cressington) for 100 seconds prior to imaging using a scanning electron microscope (XL-30, Philips) at 10kV.

Cell culture. Adipose-derived human mesenchymal stem cells (AD-hMSCs) were purchased from Lonza, which were isolated from a 42-year-old female who gave consent for the aspiration of AD-hMSCs. Ethical committee approval from the ethical advisory board of Maastricht University was given for the experimental use of AD-hMSCs. Basic medium for AD-hMSCs consisted of MEM Alpha GlutaMAX (Gibco), fetal bovine serum (FBS, 10% v/v), ascorbic-acid-2-phosphate (ASAP, 0.2 mM), and penicillin/streptomycin (10 U mL^{-1}). Cells were grown at 37 °C in a humid

atmosphere at 5% CO_2. For experimental purposes, cells were seeded on flat and topographical surfaces at a density of 10000 cells/cm^2 unless stated otherwise.

Fluorescent imaging. After cell culture, cells were washed with phosphate-buffered saline (PBS, Sigma-Aldrich) before fixation in formaldehyde (3.6% v/v) for 10 min at 37°C. After, cells were washed three times with PBS and permeabilized by the addition of Triton-X-100 (0.1% v/v) in PBS for 10 min. Following this, samples were blocked using serum (1:100) in PBT (0.02% Triton-X-100, PBS, and 0.6% bovine serum albumin) for 1hr. After another washing step, samples were incubated for 1 h in Phalloidin with a fluorochrome attached to visualize F-actin (1:500, ThermoFisher). Finally, Hoechst 33258 (1:1000, ThermoFisher) was used for the visualization of nuclei. After three subsequent washing steps, the samples were mounted in Mowiol.

MSC osteogenic differentiation and Alizarin Red quantification. Differentiation of AD-hMSCs towards the osteogenic lineage was achieved by seeding AD-hMSCs at a density of 5000 cells/cm^2. After 24h, the medium was changed with either a control or mineralization medium. The mineralization media is based on basic media supplemented with 10 nM dexamethasone (Sigma) and 10 mM β-glycerol phosphate (Sigma), while the control medium included the same components except for dexamethasone. The media was refreshed every 2-3 days, and after 21-23 days, cells were fixed overnight at 4 °C with 4% formaldehyde (VWR) in PBS. Afterward, osteogenesis was assessed through staining mineralization through a 2% Alizarin Red solution (pH=4.2) for 2 min. Excess staining was washed off with demineralized water. Alizarin Red levels were quantified through CellProfiler 3.1.8 by dividing the total red intensity levels of each image with that of the cyan intensity levels.

MSC adipogenic differentiation and Oil Red O quantification. Differentiation of AD-hMSCs towards the adipogenic lineage was achieved by seeding AD-hMSCs at a density of 15000 cells/cm^2. After 24h, the medium was replaced with either a control or adipogenic medium. The adipogenic media consist of basic media supplemented with 0.2 mM indomethacin (Sigma), 0.5 mM 3-isobutyl-1-methylxanthine (Sigma), 1 μM dexamethasone (Sigma), and 10 μg/mL Insulin (Sigma),

while the control medium consisted solely of basic media. Media was refreshed every 2-3 days, and after 21-23 days, cells were fixed overnight at 4 °C with 3.7% formaldehyde (VWR), 0.01 g/ml $CaCl_2.2H_2O$ (Merck) in PBS. Afterward, adipogenesis was assessed by rinsing the fixation solution with demineralized water and incubating the substrates in a 60% (v/v) 2-propanol (VWR) for 5 min. Then, fat droplets were stained through a freshly filtered solution of 0.3 % (w/v) Oil Red O dissolved in 60% (v/v) 2-propanol (VWR). Excess staining was washed off with demineralized water. Oil Red O levels were quantified by measuring the area covered by the droplets through Cellprofiler 3.1.8. For this, arbitrary thresholds were applied in the green and blue channels of each image.

Human mesenchymal stem cell morphology analysis. Fixed and stained samples were inverted, and fluorescent images were acquired through the glass coverslip using a fully automated Nikon Eclipse Ti-U microscope in combination with an Andor Zyla5.5 four megapixels camera. Fluorescent images were analyzed through CellProfiler 3.1.8 [49], applying custom-made pipelines. All images were cropped in order to remove out-of-focus objects. Objects touching the border of the subsequent images were filtered out of the dataset. After illumination corrections, the morphology of the nucleus was captured by the Otsu adaptive thresholding method applied on the Hoechst 33258 image channel. Subsequently, cell morphology was determined by applying propagation and Otsu adaptive thresholding on the Phalloidin image channel. Mis segmentation artifacts were removed by applying an arbitrary threshold on nuclei and cell size. For visualization purposes, we enhanced the brightness and contrast of the images representing cellular morphologies. The imaging software Fiji was used for image visualization.[70]

Bacteria imaging and data acquisition. *Pseudomonas aeruginosa* PAO1 was chosen to test the performance of natural topography replicas against bacterial surface attachment. PAO1 was routinely grown at 37°C in lysogeny broth (LB) or LB agar. Tryptic soy broth (TSB) supplemented with human serum (10% v/v) was used as the growth medium for bacterial attachment assays. Prior to incubation with bacteria, topographies were washed by dipping in distilled water and sterilized in

ethanol (70% v/v). The air-dried samples were placed in Petri dishes (60 mm x 13 mm) and incubated statically at 37°C in 10 ml of growth medium inoculated with diluted (optical density: OD600 nm = 0.01) bacteria from overnight cultures. After 4h incubation, topography samples were removed and washed in PBS (pH 7.4) to remove loosely attached bacteria. After rinsing with distilled water, attached bacteria were stained with SYTO9 (50 µM; Molecular Probes, Life Technologies) for 30 min at room temperature. After staining, topographies were rinsed with distilled water, air-dried, and mounted on a glass slide using Prolong antifade reagent (Life Technologies). Topographies were then imaged by confocal laser scanning using a Zeiss LSM 700 microscope (Carl Zeiss, Germany) and 488 nm laser as light source. Since the bacterial cells may attach at different heights on the micro-patterns, images were initially acquired as ~75 µm range Z-stacks (1.5 µm steps - 50 slides) from the TUs using a 10x objective (Zeiss, EC Plan-Neofluar 10x/0.30 Ph 1).

PCA analysis. Greyscale images of the natural surfaces were captured through profilometric imaging through a Keyence VK-H1XM-131 profilometer. Height profiles were represented as grayscale images, where black represented the bottom of the surfaces and white top. For artificial surfaces, pixel values 0 correspond to the bottom and 10 to the top of the pillars, which matches their height in µm. Since the natural surfaces height profile gradually varied from the bottom to the top of the surface, the pixel values also were counted and were arranged in a range from 0 (bottom) to the height value as measured by profilometry. As the input for the algorithm, we have used pixel values directly, 250000 values per surface. The resulting images contained information about the height of the elements as well as their structure on the area of 200x200 µm with resolution 500x500 pixels. Profilometry software was further used to correct for sample tiltness. We further corrected images by applying local contrast enhancement algorithm as implemented in sci-kit Python 3.7.3 package. Intensity values in the image were normalized further by subtracting minimum pixel value and dividing by max pixel intensity value per surface. The final image was obtained by multiplying a resulting matrix by height value as measured with the profilometer.

Texture quantification. Spatial distribution of bacteria attachment on the TopoChip and natural surfaces was quantified by image texture features, which were extracted from the thresholded images. Image analysis was performed in CellProfiler 3.1.8.

Texture Feature Selection. To distinguish texture features that were the most discriminative between TopoChip and natural surfaces, we employed a binary classification algorithm. Particularly we trained the Extreme Gradient Boosting (EGB) model on a hold-out subset that contained over 500 images of the bacteria attachment on either TopoChip or natural surfaces. The accuracy of the trained model was validated using the training set, and the accuracy value was 0.98. An accuracy of 1.0 corresponds to a perfectly performing classification algorithm. We further quantified the importance of the features with the Mean Decrease Accuracy (MDA) algorithm that measures how model accuracy decreases when a feature is excluded from the prediction.

Spatial relationship features. We identified that the texture feature 'InfoMeas1 for scales 64 and 2' was the most important for distinguishing bacteria attachment between TopoChip and natural surfaces. 'InfoMeas1' is a measure of the total amount of information contained within a region of pixels derived from the recurring spatial relationship between specific intensity values.[71]. We rename them in the text and Figure 3e as Spatial Relationship Feature 1 (SRF1) and Spatial Relationship Feature 2 (SRF2). For visualization purposes, we transformed SRF1 by taking its cubic root. The scatter plot in Figure 3f represents 100 randomly selected surfaces from both TopoChip and natural groups.

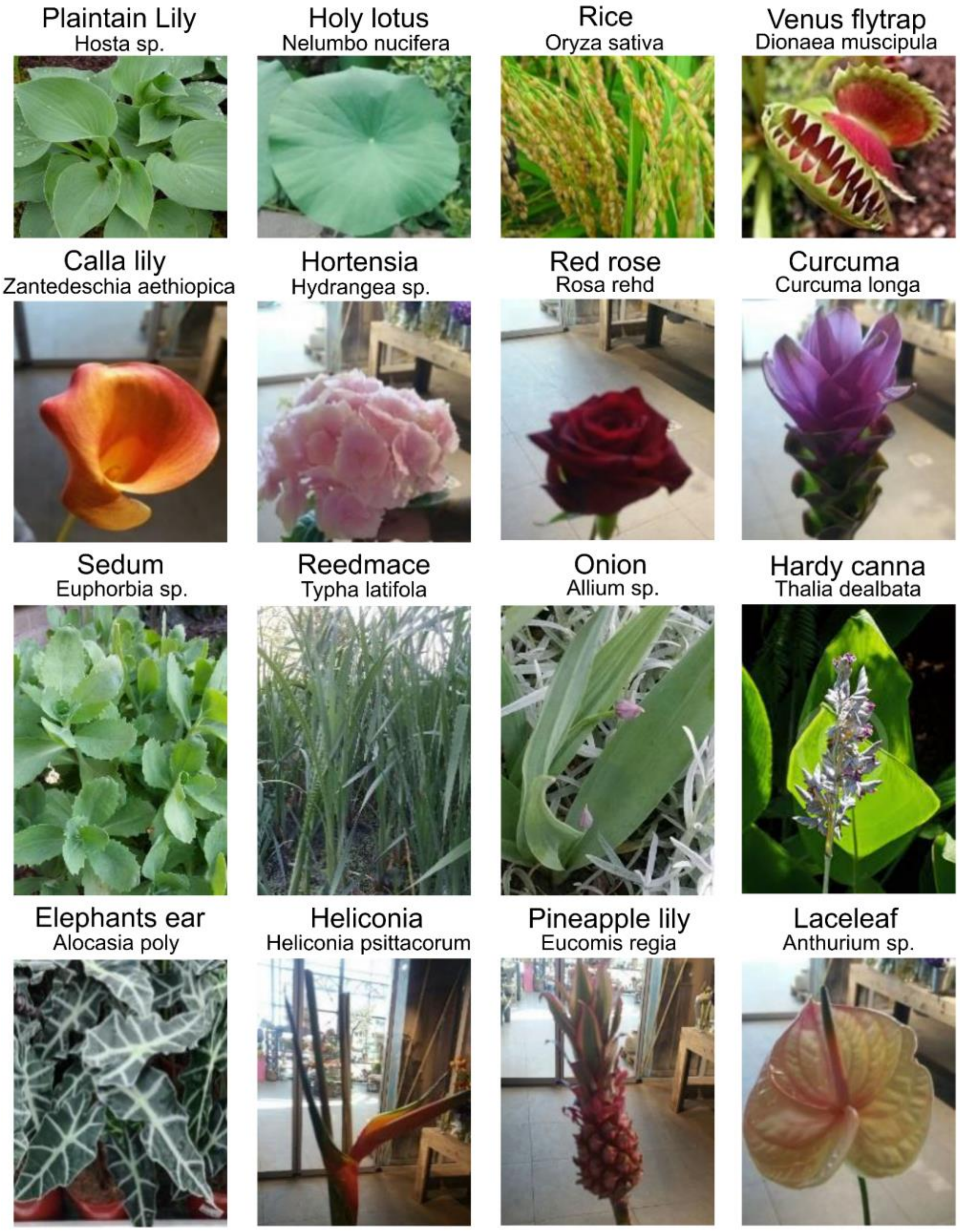

Supplementary Figure 1. Photographs of the sixteen plants used to obtain PS replicas of their leaf's topography.

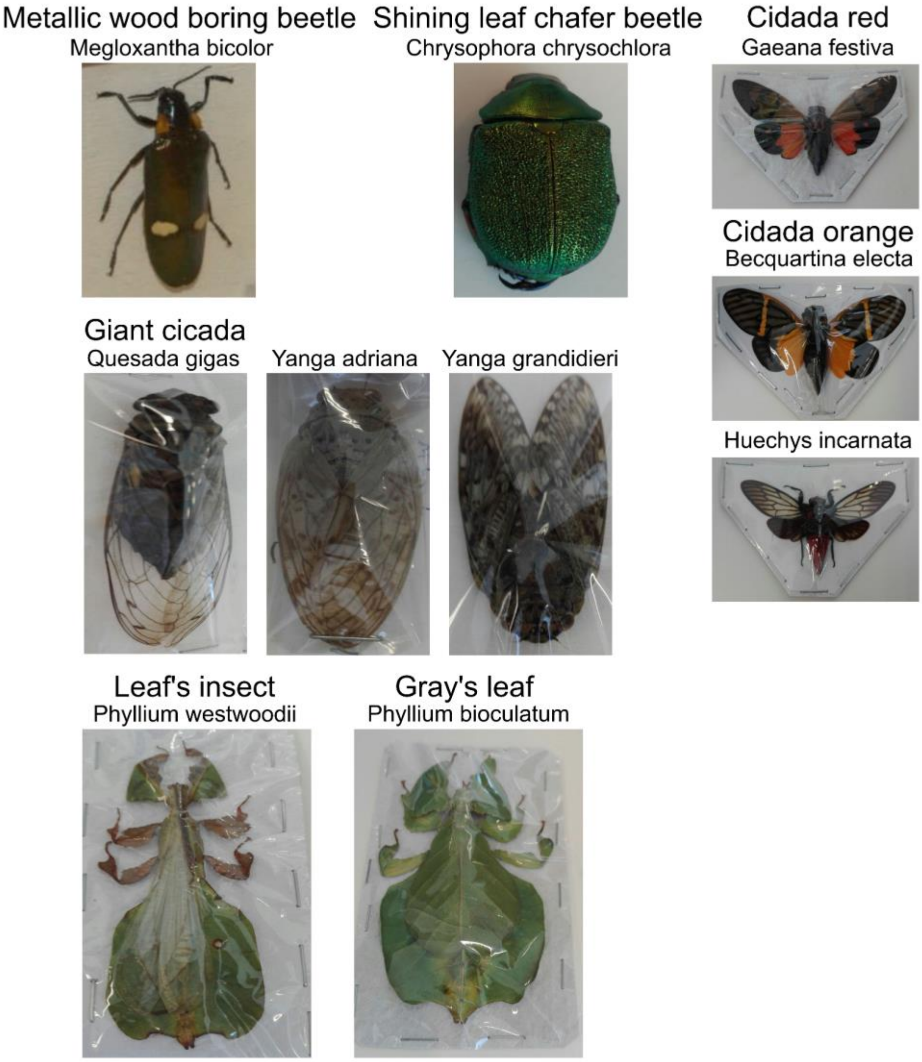

Supplementary Figure 2. Photographs of the ten insects used to obtain PS replicas of their wings.

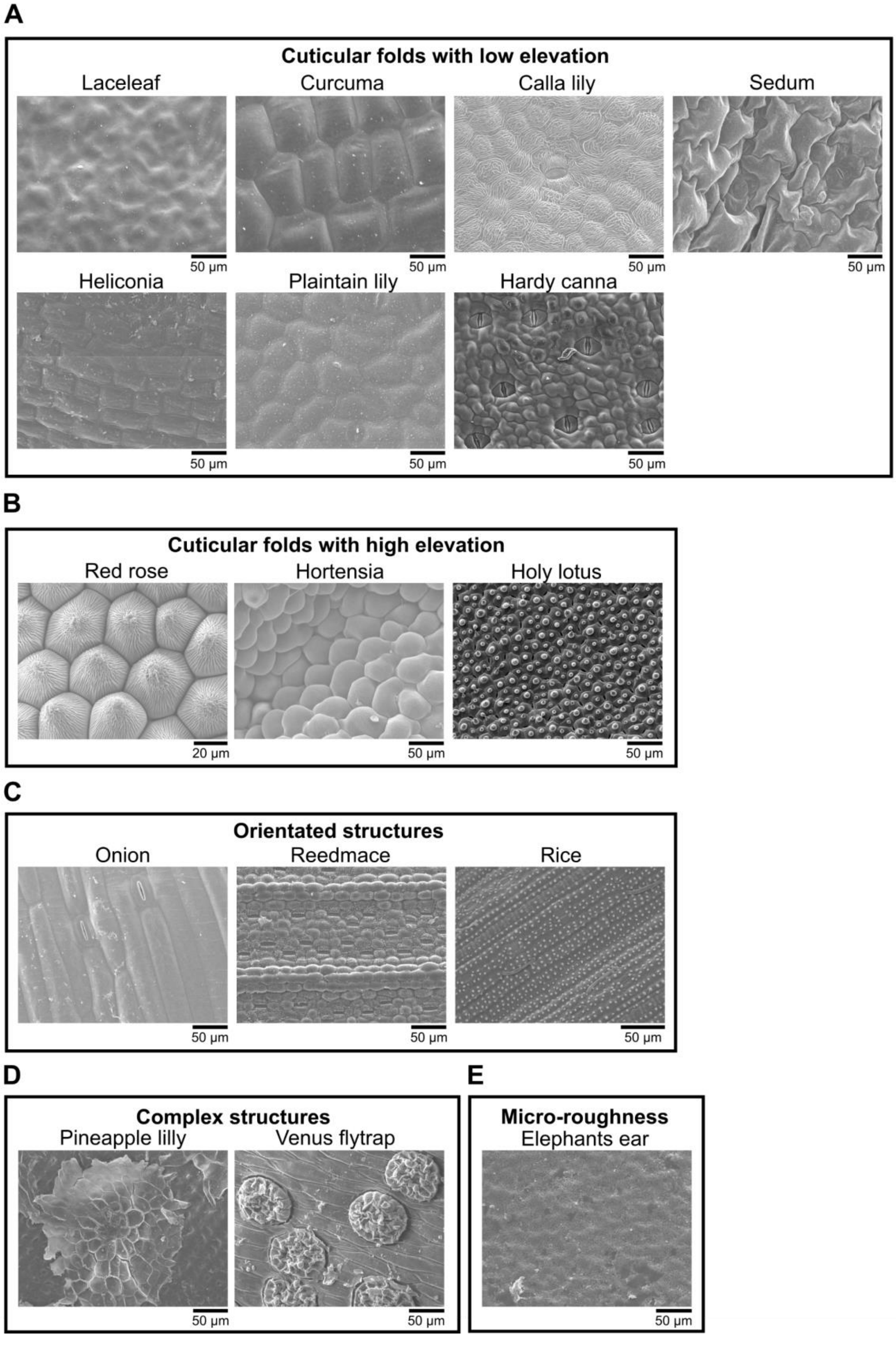

Supplementary Figure 3. SEM images of plant surfaces revealed a broad diversity in surface topography. **A)** Structures with low elevation are present on the Laceleaf, Calla lily, Curcuma, Sedum, Heliconia, Plantain lily, and Hardy canna. **B)** Cuticular folds with complex and hierarchical structures are found on the Red rose, Hortensia, and Holy lotus. **C)** Oriented structures are found on the Onion, Reedmace, and Rice. **D)** Complex structures are found on the Pineapple lilly and Venus flytrap. **E)** Crystalline waxes present on the Elephants Ear give rise to micro-roughness structures.

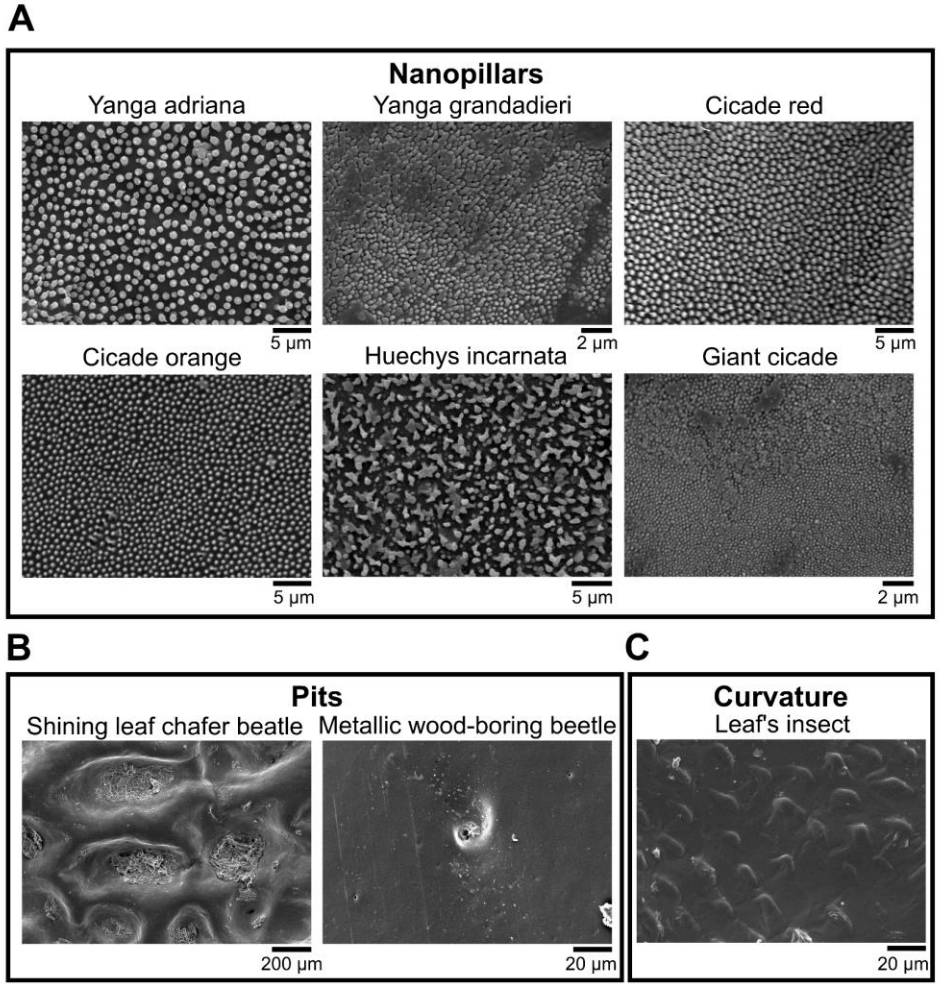

Supplementary Figure 4. SEM images of insect surfaces expanded the design space with nanotopographies, wells, and curvature. **A)** Nanopillars with different dimensions are found on different cicada species. **B)** Beetles exhibit pit-like structures on their surface, as shown for the Shining leaf chafer beetle and the metallic-wood boring beetle. **C)** Surfaces containing curvature are found on insects resembling leaves of plants, as shown for the Leaf’s insect.

A

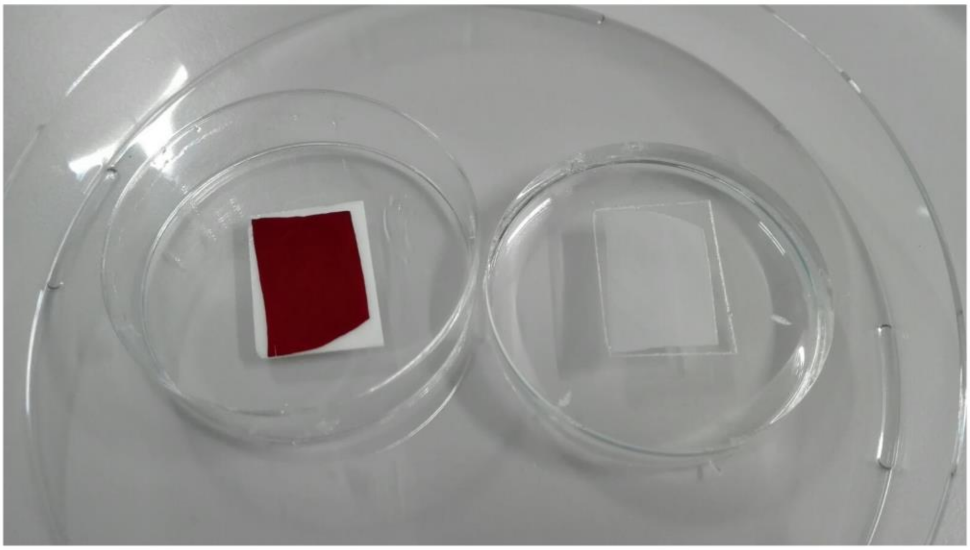

B

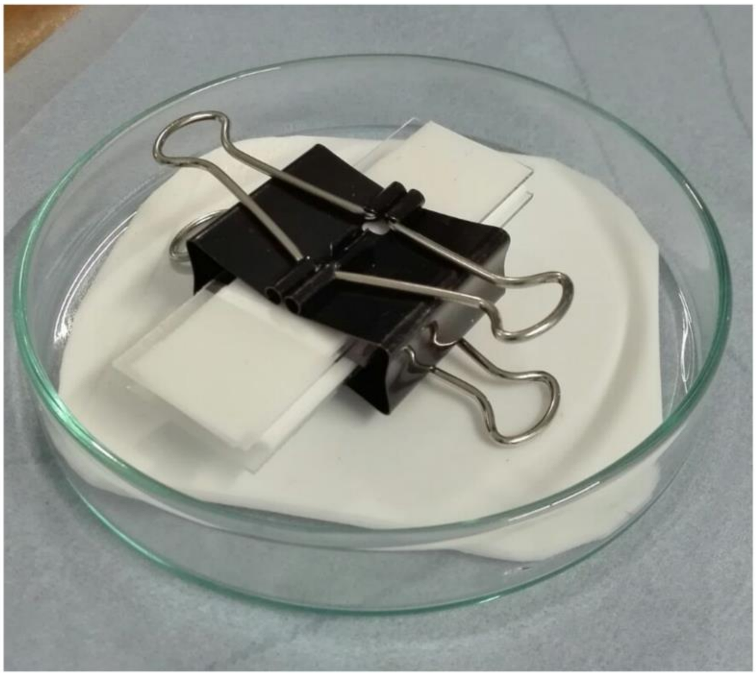

Supplementary Figure 5. Easy 'sandwich' imprinting fabrication. **A)** PDMS is poured on the Red rose imprint, after which it is peeled-off after curing for 24h. **B)** Afterwards, Teflon sheets, PDMS, and PS are sandwiched and pressured together with binders before placing the 'sandwich' in the oven.

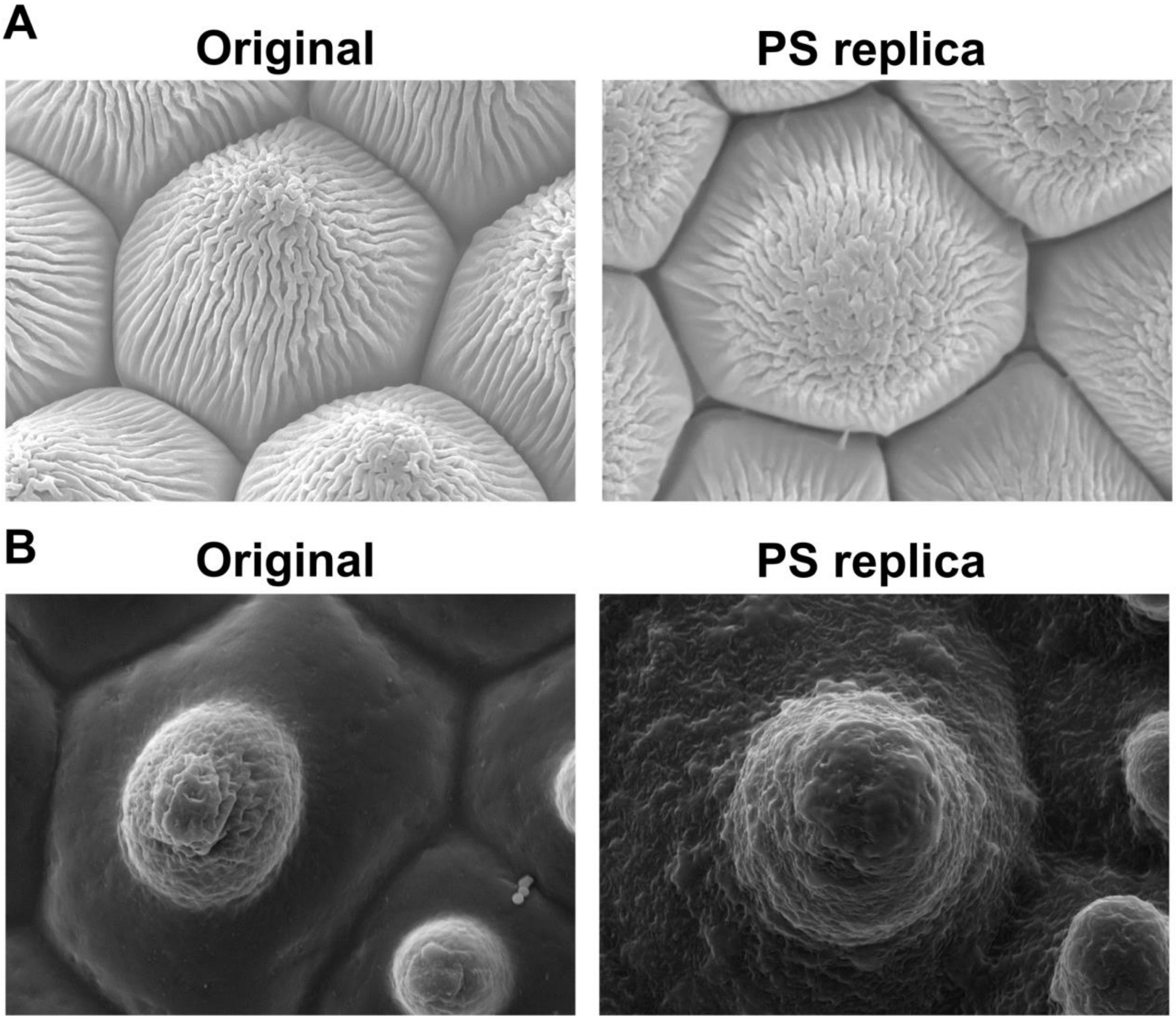

Supplementary Figure 6. A) SEM images of the Red rose. The Red rose topography can be transferred with high fidelity to polystyrene. **B)** SEM images of the Holy lotus. The Holy lotus topography can be transferred with high fidelity to polystyrene, yet with an increase in surface roughness levels.

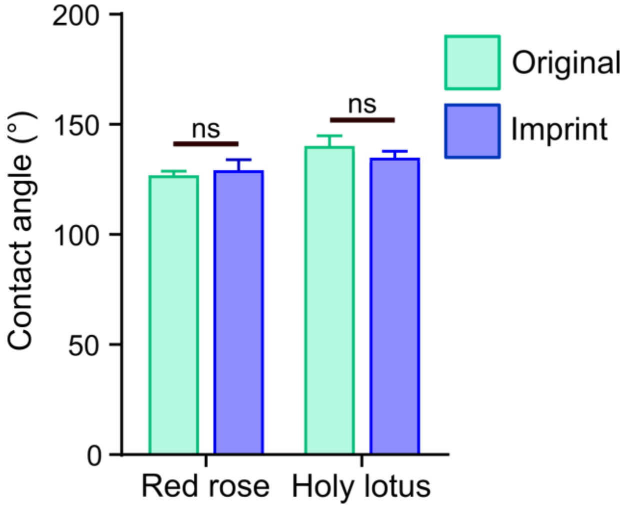

Supplementary Figure 7. No difference in the water contact angle was observed between the original surface and the PS imprints of the Red rose and Holy lotus.

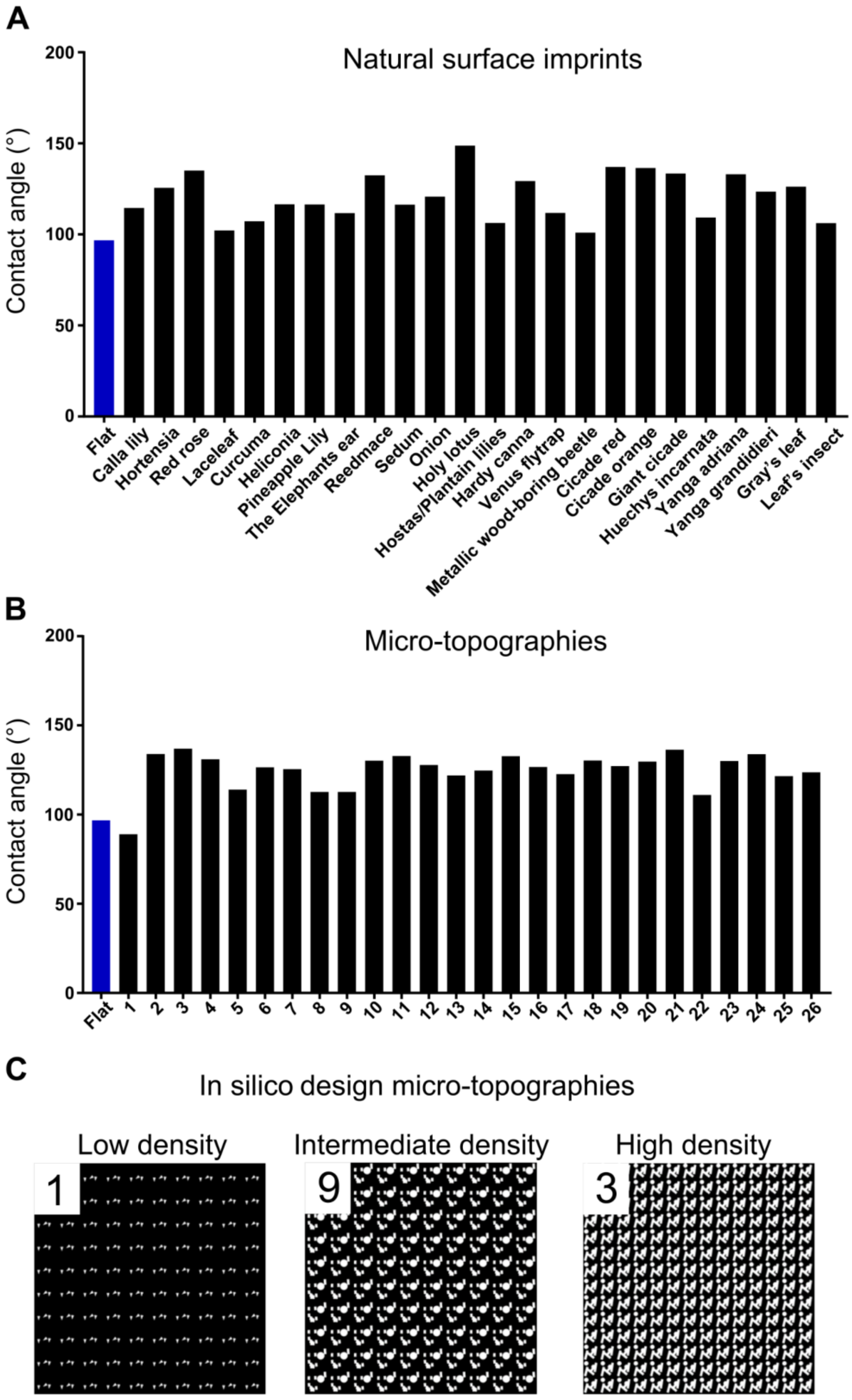

Supplementary Figure 8. Water contact angles on artificial and natural topographies. a) On the natural topographies, we found that the water contact angle on the Holy lotus was highest. **b)** Water contact angle is roughly uniform throughout artificial micro-topographies. **c)** The water contact angle of representative artificial micro-topographies was lowest on topographies with low pattern density (1), while the water contact angle was highest on surfaces with high pattern density (3). The number of the micro-topography in the top right corner (1, 9, and 3) corresponds to the x-axis in **b)**.
